# RXR Expression Profiles in Yak Reproductive Tissues During Follicular, Luteal, and Pregnancy Phases

**DOI:** 10.3390/ani15192814

**Published:** 2025-09-26

**Authors:** Xiaokun Zhang, Wenbin Ma, Xin Ma, Jianying Chang, Juan Yang, Meng Wang, Libin Wang, Qian Zhang, Yangyang Pan

**Affiliations:** College of Veterinary Medicine, Gansu Agricultural University, Lanzhou 730070, China; 15983106250@163.com (X.Z.); mwb9985@126.com (W.M.); mxinmaxin@163.com (X.M.); changjy2000@163.com (J.C.); yang20250615@163.com (J.Y.); wangmeng@gsau.edu.cn (M.W.); wanglb@gsau.edu.cn (L.W.); zq880204@126.com (Q.Z.)

**Keywords:** estrous cycle, reproductive physiology, RXRs, yak, gestation

## Abstract

The yak is a large ruminant that lives in the high-altitude and hypoxic environment of the Qinghai–Tibet Plateau in China and typically exhibits limited reproductive capacity, posing a significant challenge to the advancement of animal husbandry in the region. Retinoid X receptors (RXRs), as an important member of the NR superfamily, play a key role in the regulation of reproductive hormone synthesis, follicular development, and embryo implantation. However, there is still a lack of systematic research on the expression characteristics and potential functions of RXRs in the yak’s reproductive system. This study characterized RXR expression in ovarian, uterine, and oviductal tissues from three yaks per reproductive phase (follicular, luteal, and pregnancy). Using Quantitative Real-Time PCR Experiments (RT-qPCR), Western blot (WB), immunohistochemistry (IHC), and immunofluorescence (IF), we analyzed RXR mRNA and protein expression and localization. RXR expression varied significantly (*p* ≤ 0.05), peaking in ovaries during the follicular phase, oviducts during the luteal phase, and uteri during pregnancy. RXRs were localized in ovarian granulosa and theca cells, oviductal epithelium, and uterine endometrial glands, with dynamic nuclear–cytoplasmic shifts. These findings suggest RXRs regulate key reproductive processes in yaks, offering insights on improving fertility in high-altitude environments.

## Simple Summary

Retinol X receptors (RXRs), including α, β, and γ subtypes, play a key role in the reproductive process and are involved in regulating various physiological processes, including gonadal development, sexual differentiation, reproductive behavior, and reproductive-related diseases. Yaks (*Bos grunniens*), adapted to the high-altitude Qinghai–Tibet Plateau, exhibit low fertility due to environmental stressors, impacting livestock farming. In this study, we investigated the RXR gene expression profiles in the uterine, ovarian, and oviductal tissues of yaks during distinct reproductive phases. The results indicated that there are significant differences in the expression of RXRs in the ovaries, oviducts, and uterus of yaks during various reproductive phases. Our results indicate that RXRs may be involved in a wide range of yak reproductive stages, namely, follicular development, fertilization, and early embryonic development. This observation provides a preliminary framework for investigating the influence of these receptors on the reproductive physiology of yaks; although, it is important to note that this framework is not definitive.

## 1. Introduction

For yaks, a species uniquely adapted to China’s high-altitude plateaus and a linchpin for local farmers’ livelihoods, low reproductive rates stand as a major roadblock to husbandry [1,2]. The plateau’s harsh climate shapes their distinctive reproductive physiology. Yaks have an average lifespan of 20.57 ± 0.39 years, a body weight ranging from 200 to 650 kg, and an age at first estrus of 2.1 to 2.9 years. Their estrous cycle lasts 21.1 ± 0.29 days, the estrous duration is 16.9 ± 0.7 h, and the gestation period is 259.73 ± 1.48 days [3]. RXRs, integral to the NR superfamily, are vital transcription factors in various mammalian biological processes. They consist of three subtypes, RXRα, RXRβ, and RXRγ, resembling other nuclear receptors in domain structure A/B, C, D, E, and F [4].

RXRs exert their effects by forming homodimers, homotetramers, and heterodimers with a wide range of nuclear receptors—such as PPARs, LXRs, VDR, and RARs —shuttling between the cytoplasm and nucleus to regulate processes such as cell differentiation, development, metabolism, immune responses, cell proliferation, estrus regulation, and early embryonic development [5].

Mangelsdorf and colleagues first highlighted RXRs in 1992 as nuclear receptors responsive to vitamin A derivatives; subsequently, they have been found in tissues and organs across a range of mammals, underscoring their versatility [6]. A wealth of studies highlights RXRs’ vital roles in maintaining skin homeostasis, regulating glucose–lipid metabolism, and driving bone remodeling [7,8,9]. In reproductive physiology, RXRs prove indispensable for post-implantation embryonic development in mice; knocking out RXRα triggers embryonic death between days 12.5 and 16.5, marked by vitamin deficiency syndromes; in comparison, RXRα/RXRβ knockouts lead to even earlier lethality and flaws in chorioallantois placenta formation [10]. However, mice with RXRβ/RXRγ mutations survive with no related issues [11]. In the study by Li et al. [12], the authors also revealed RXRs’ role in the upstream gene regulation of seasonal estrus in sheep. RXRs regulate oocyte maturation through classic vitamin A pathways, promoting meiosis [13]. In addition, they support immune tolerance in the testes via FASL/FAS/Caspase8 pathways [14]. RXRs likely play crucial roles in estrus, follicular development, and early embryogenesis [15,16].

While RXRs are known to regulate reproductive physiology in other species, their specific roles in yak ovaries, oviducts, and uteri remain unexplored, representing a critical research gap. We hypothesize that RXR expression varies across follicular, luteal, and pregnancy phases in yak reproductive tissues, reflecting their roles in regulating fertility. In this study, we examine RXR expression and localization in yak reproductive tissues across follicular, luteal, and pregnancy phases, using RT-qPCR, Western blot analysis, immunohistochemistry, and immunofluorescence to lay a solid foundation for understanding RXRs’ contributions to yak reproduction and addressing fertility challenges on the plateau.

## 2. Materials and Methods

### 2.1. Sample Collection

Samples were collected from Xining slaughterhouse in Qinghai Province in September, where the average ambient temperature ranges from 10 to 12 °C, and the relative humidity is around 65 to 70%. Healthy female yaks at three physiological stages (follicular phase, luteal phase, and pregnancy) were chosen due to challenges in selecting yaks at various physiological stages and ethical concerns. Each stage included three yaks. All yaks were multiparous individuals. The yaks were adult females, aged 3–5 years, with body weights of 300–350 kg. After being sacrificed via arterial bloodletting, ovarian, oviduct, and uterine tissue samples were rapidly collected during the follicular phase, luteal phase, and pregnancy. The criteria for judging the follicular phase was the presence of the tertiary follicle with a diameter of 5–10 mm on the ovary; the criterion of the luteal phase was the presence of a clear corpus luteum on the ovary, with a diameter of 15–20 mm, and that the blood vessels were rich; the criterion for pregnancy was the presence of a fetus in the uterus, with the length of the fetal head and arm being 10–20 cm, and the pregnancy time point being 2–4 months. The tissue samples used for immunohistochemistry and immunoassay analysis were rinsed with normal saline and stored in 4% paraformaldehyde fixative. After one month of fixation at room temperature, the samples were dehydrated and embedded in paraffin blocks. The samples for RT-qPCR and Western blot analysis were promptly wrapped in aluminum foil and submerged in liquid nitrogen for preservation, subsequently stored in a freezer at −80 °C to maintain RNA and protein integrity. Subsequent experiments were conducted within one month to validate the integrity of the samples. To enhance reliability, multiple tissue samples (n = 3 per tissue per yak) were analyzed.

### 2.2. Key Instruments and Reagents

The major instruments used in this study included a conventional PCR system (Eppendorf, Hamburg, Germany), a tissue embedding station and rotary microtome (Leica, Wetzlar, Germany), a DP73 microscope digital camera (Olympus, Tokyo, Japan) Key reagents comprised Trizol reagent (TransGen Biotech, Beijing, China), RIPA lysis buffer (TransGen Biotech, Beijing, China), 4× protein loading buffer (Solarbio, Beijing, China), DAPI staining solution (Solarbio, Beijing, China), an SP immunohistochemistry kit (Solarbio, Beijing, China). Primary antibodies used included rabbit polyclonal antibodies against RXRα, RXRβ, and RXRγ. (Affinity Biosciences, Cincinnati, OH, USA)

### 2.3. Detection of RXRα, RXRβ, and RXRγ Expression Levels in Yak Ovaries, Uteri, and Fallopian Tubes Across Reproductive Phases

#### 2.3.1. mRNA Expression Levels of RXRα, RXRβ, and RXRγ Across Reproductive Phases

To examine the mRNA expression levels of RXR in the ovary, uterus, and oviduct during different reproductive cycle phases, total RNA was extracted from these ovarian, oviduct, and uterine tissues at different stages using a transzol kit, and after determining its concentration and purity, the total RNA that reached the concentration and purity standard was reverse-transcribed into cDNA. Drawing on GenBank sequences for Bos taurus—RXRα, RXRβ, and RXRγ—primers were developed with Primer 6.0, using β-actin as the reference gene, synthesized by Shanghai Sangon, with the primer information displayed in Table 1. Reactions were performed on a LightCycler 96 real-time quantitative PCR system using 2× SYBR Green Premix, following the MIQE guidelines [17]. Each 20 μL reaction contained 1 μL cDNA, 0.4 μL of each primer (10 μM), 10 μL 2× qPCR SuperMix, and 8.2 μL dd water. Four biological replicates were established in each group. The relative expression was calculated based on a previously described method. The cycling conditions are as follows: the sample is heated to 94 °C for 30 s, followed by 40 cycles of heating to 94 °C for 5 s, cooling to 60 °C for 30 s, heating to 72 °C for 15 s, and a final extension at 72 °C for 5 min. Amplicon specificity was confirmed by melting curve analysis results. The 2^−ΔΔCt^ method was used to calculate relative expression, and β-actin was the reference gene. All qPCR data were generated in compliance with MIQE guidelines.

#### 2.3.2. Protein Expression Analysis of RXRα, RXRβ, and RXRγ in Yak Ovaries, Uteri, and Oviducts Across Reproductive Phases

Tissue samples across yak reproductive stages were first pre-chilled in liquid nitrogen, put in a mill, and ground to a granular state; then, they were mixed with protein lysis buffer and PMSF protease inhibitor, shaken on ice for 2 h, and centrifuged at 4 °C to collect the supernatant for analysis. This supernatant was blended 3:1 with protein loading buffer, vigorously mixed, denatured in a 100 °C metal bath for 15 min, cooled to room temperature, and stored at −20 °C.

The protein samples were loaded, separated by sodium dodecyl sulfate-polyacrylamide gel electrophoresis, and electrotransferred onto the PVDF membrane. Membranes were blocked with 5% skim milk at room temperature for 3 h, probed with RXR anti-bodies (1:4000 for RXRα, 1:3000 for RXRβ, 1:5000 for RXRγ) overnight at 4 °C, washed thrice for 20 min with PBST, incubated with secondary antibodies in a shaker for 40 min, washed again, and treated with a chemiluminescent solution. Bands were scanned using a luminometer and their intensities measured and normalized in ImageJ 6.0.

### 2.4. Distribution Detection of RXRα, RXRβ, and RXRγ in Yak Ovaries, Uteri, and Oviducts Across Reproductive Phases

IHC and IF staining was used to evaluate and localize RXRα, RXRβ, and RXRγ in yak ovarian, uterine, and oviductal tissues during follicular, luteal, and pregnant phases. Fixed tissue samples were carefully dehydrated, cleared, and embedded into paraffin blocks, and then sliced into 4 μm sections using the Leica microtome. After baking at 60 °C for 6 h, samples underwent deparaffinization and rehydration, followed by antigen retrieval via microwave heating in citrate buffer, cooling naturally to room temperature. For IHC, following the SP kit protocol, tissues were blocked, sealed, and incubated with RXR antibodies (1:200 for RXRα and RXRβ, 1:300 for RXRγ) overnight at 4 °C; the negative control group was dripped with PBS of the same volume, and then treated with secondary and tertiary antibodies, developed with DAB, counterstained with hematoxylin, briefly treated with acid alcohol, rinsed, dehydrated, cleared, mounted with neutral resin, and photographed under a microscope. For ovarian tissues in the follicular phase, the visual fields containing secondary follicles were selected for photography.

For IF staining, tissue sections were permeabilized with 0.5% Triton X-100 following blocking. Subsequently, sections were incubated with 5% BSA for 2 h and then with RXR antibodies overnight at 4 °C. The negative control group was dripped with PBS of the same volume. Fluorescent secondary antibodies were added for 1.5 h at 37 °C. The nuclei were counterstained with DAPI. The acquisition of digital images was conducted using a fluorescence inverted microscope. For ovarian tissues in the follicular phase, visual fields containing secondary follicles were selected for image capture.

### 2.5. Statistical Analysis

All data are expressed as the mean ± standard error (Mean ± SEM) of three independent experiments. The data were tested for normality using the Shapiro–Wilk test in SPSS 27.0 software, confirming normal distribution (*p* > 0.05 for all groups). One-way ANOVA was used to compare RXR expression across reproductive phases, followed by Tukey’s post hoc test to identify specific group differences. All statistical analyses were performed using GraphPad Prism 6.0 software. The difference in mRNA and protein expression levels was considered statistically significant at a *p*-value ≤ 0.05. Despite the limited sample size (n = 3 per stage), statistical robustness was supported by low variability (SE < 10%) and consistent results across multiple assays.

## 3. Results

### 3.1. RXRα, RXRβ, and RXRγ Expression in Yak Ovaries, Uteri, and Oviducts Across Reproductive Phases

#### 3.1.1. Comparison of *RXRα*, *RXRβ*, and *RXRγ* mRNA Relative Expression Across Reproductive Phases

**Figure 1:**
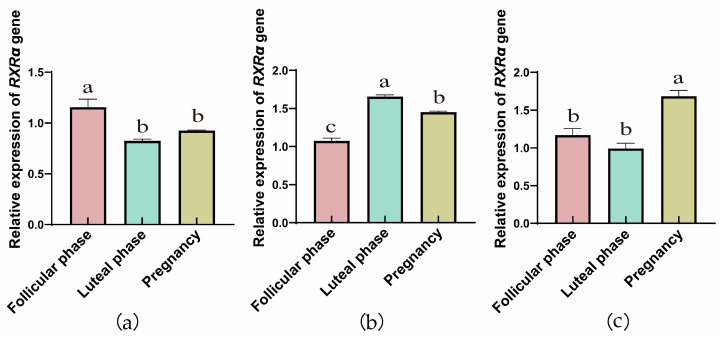
RT-qPCR analysis of *RXRα* gene expression in yak ovaries (**a**), oviducts (**b**), and uteri (**c**) across different reproductive phases (the different letters indicate that the values are significantly different at *p* ≤ 0.05).

**Figure 2:**
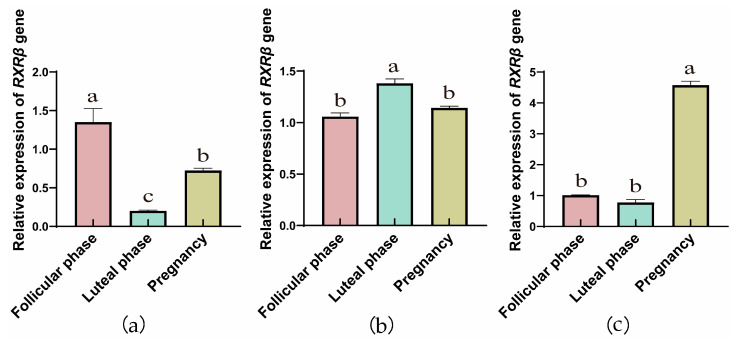
RT-qPCR analysis of *RXRβ* gene expression in yak ovaries (**a**), oviducts (**b**), and uteri (**c**) across different reproductive phases (the different letters indicate that the values are significantly different at *p* ≤ 0.05).

**Figure 3:**
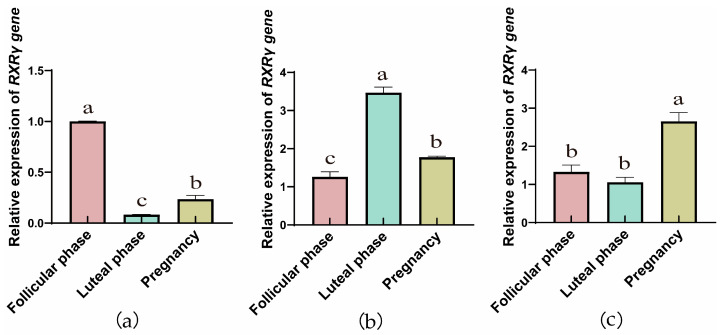
RT-qPCR analysis of *RXRγ* gene expression in yak ovaries (**a**), oviducts (**b**), and uteri (**c**) across different reproductive phases (the different letters indicate that the values are significantly different at *p* ≤ 0.05).

The RT-qPCR results showed that the genes of RXRs were expressed in yak ovaries, oviducts, and uteri across the follicular, luteal, and gestational stages. In ovarian tissue (Figure 1a, Figure 2a and Figure 3a), the *RXRα*, *RXRβ*, and *RXRγ* genes exhibit a trend of initial decrease followed by an increase. However, in the fallopian tube, the relative expression of the *RXRα*, *RXRβ*, and *RXRγ* genes exhibit a trend of initial increase followed by a decrease. In addition, with the progression of reproductive stages, the relative expression levels of *RXRα*, *RXRβ*, and *RXRγ* genes in the uterus exhibit a trend of initial decrease followed by an increase.

#### 3.1.2. Comparing RXRα, RXRβ, and RXRγ Protein Levels Across Yak Reproductive Phases

**Figure 4:**
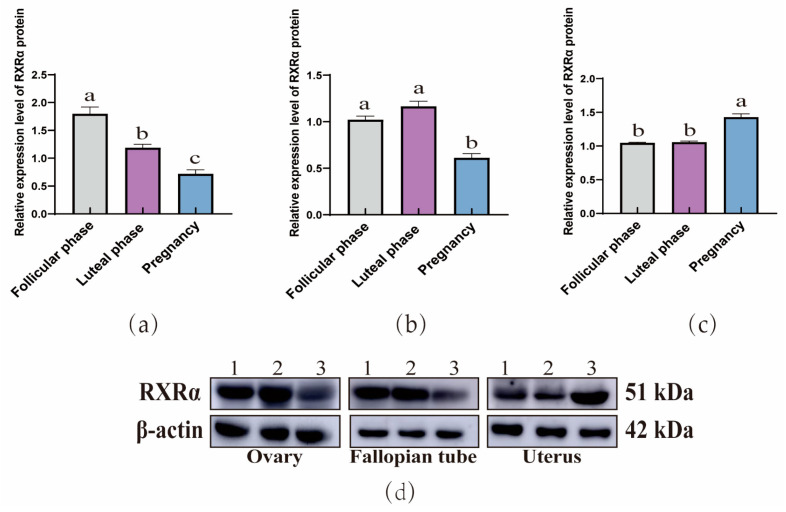
Western blot analysis of the RXRα protein expression in yak ovaries (**a**), oviducts (**b**), and uteri (**c**) across different reproductive phases. (**d**) β-actin and the RXRα protein in ovaries, oviducts, and uteri during different stages of the reproductive cycle. 1: Follicular phase; 2: Luteal phase; 3: Pregnancy phase (the different letters indicate that the values are significantly different at *p* ≤ 0.05).

**Figure 5:**
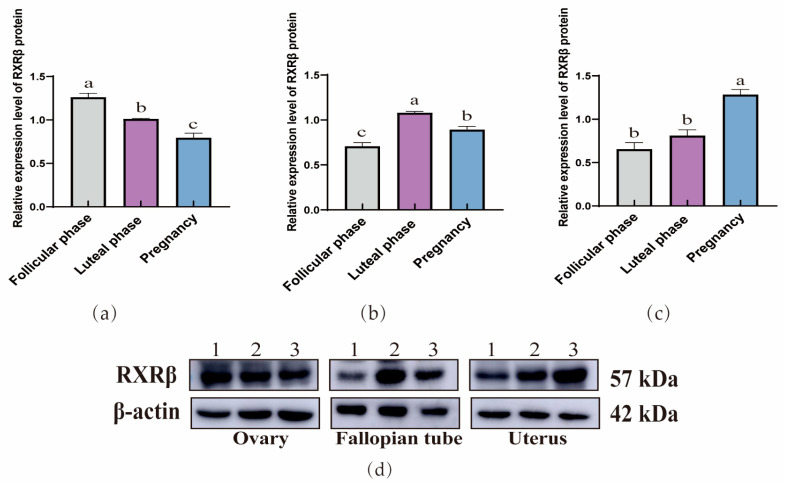
Western blot analysis of the RXRβ protein expression in yak ovaries (**a**), oviducts (**b**), and uteri (**c**) across different reproductive phases. (**d**) β-actin and the RXRα protein in ovaries, oviducts, and uteri during different stages of the reproductive cycle. 1: Follicular phase; 2: Luteal phase; 3: Pregnancy phase (the different letters indicate that the values are significantly different at *p* ≤ 0.05).

**Figure 6:**
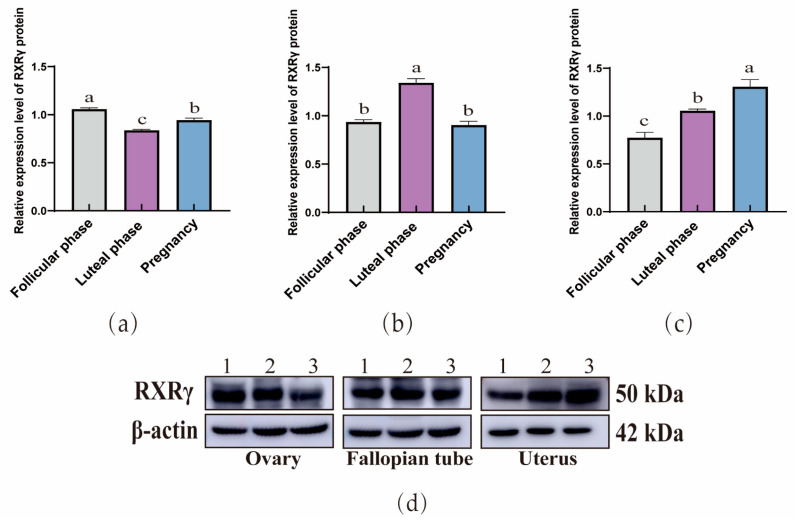
Western blot analysis of the RXRγ protein expression in yak ovaries (**a**), oviducts (**b**), and uteri (**c**) across different reproductive phases. (**d**) β-actin and the RXRα protein in ovaries, oviducts, and uteri during different stages of the reproductive cycle. 1: Follicular phase; 2: Luteal phase; 3: Pregnancy phase (the different letters indicate that the values are significantly different at *p* ≤ 0.05).

The WB results demonstrated that RXRα (Figure 4), RXRβ (Figure 5), and RXRγ (Figure 6) proteins were ubiquitous in the ovaries, oviducts, and uteri of yaks at different stages. In the ovaries, the expression of RXRα and RXRβ proteins exhibit a consistent decreasing trend, whereas RXRγ proteins display a trend of initial decrease followed by a subsequent increase. In addition, in the oviducts, the expression levels of RXRα, RXRβ, and RXRγ proteins exhibit a trend of initial increase followed by a decrease. Moreover, in the uterus, the expression levels of RXRα, RXRβ, and RXRγ proteins exhibit an increasing trend.

### 3.2. Mapping RXRα, RXRβ, and RXRγ Distribution in Yak Ovaries, Uteri, and Oviducts Across Reproductive Phases

**Figure 7:**
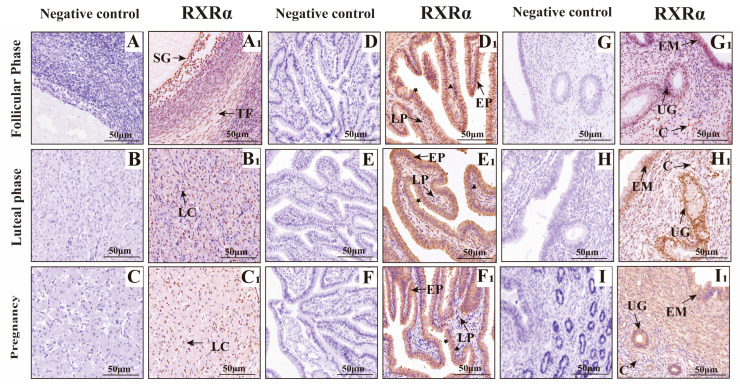
Immunohistochemical staining of RXRα in yak ovaries, oviducts, and uteri. (**A**–**I**) Negative control expression; (**A1**,**B1**,**C1**) ovaries; (**D1**,**E1**,**F1**) oviducts; (**G1**,**H1**,**I1**) uteri. Key structures include SG: follicular granulosa layer; TF: theca folliculi; LC: luteal cells; LP: stroma; EP: mucosal epithelium; EM: endometrial epithelium; UG: uterine glands; C: blood vessels; ▲: secretory cells; ★: ciliated cells. Follicular phase: groups (**A**,**D**,**G**); luteal phase: groups consisting of (**B**,**E**,**H**); pregnancy phase: groups consisting of (**C**,**F**,**I**). This figure illustrates the RXRα distribution, offering a clear picture of its presence in the reproductive tissues of yaks living on the plateau.

**Figure 8:**
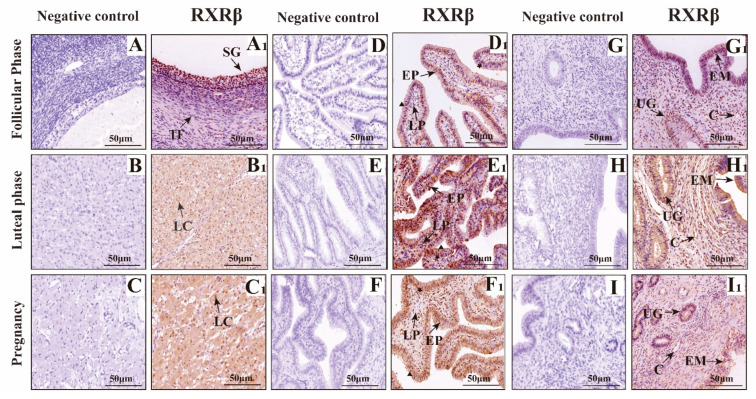
Immunohistochemical Staining of RXRβ in Yak ovaries, oviducts, and uteri. (**A**–**I**) Negative control expression; (**A1**,**B1**,**C1**) ovaries; (**D1**,**E1**,**F1**) oviducts; (**G1**,**H1**,**I1**) uteri. Key structures include SG: follicular granulosa layer; TF: theca folliculi; LC: luteal cells; LP: stroma; EP: mucosal epithelium; EM: endometrial epithelium; UG: uterine glands; C: blood vessels; ▲: secretory cells; ★: ciliated cells. Follicular phase: groups (**A**,**D**,**G**); luteal phase: groups consisting of (**B**,**E**,**H**); pregnant phase: groups consisting of (**C**,**F**,**I**). This figure showcases RXRβ distribution, offering a clear map of its presence in yak reproductive tissues on the plateau.

**Figure 9:**
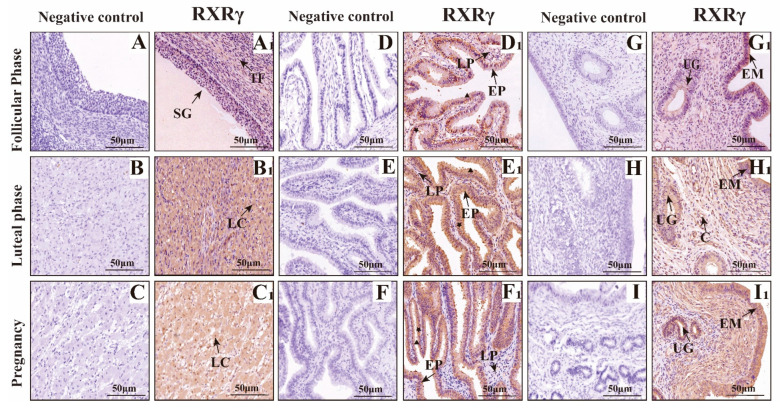
Immunohistochemical Staining of RXRγ in Yak ovaries, oviducts, and uteri. (**A**–**I**) Negative control expression; (**A1**,**B1**,**C1**) ovaries; (**D1**,**E1**,**F1**) oviducts; (**G1**,**H1**,**I1**) uteri. Key structures include SG: follicular granulosa layer; TF: theca folliculi; LC: luteal cells; LP: stroma; EP: mucosal epithelium; EM: endometrial epithelium; UG: uterine glands; C: blood vessels; ▲: secretory cells; ★: ciliated cells. Follicular phase: groups (**A**,**D**,**G**); luteal phase: groups consisting of (**B**,**E**,**H**); pregnant phase: groups consisting of (**C**,**F**,**I**). This figure showcases RXRγ distribution, offering a clear map of its presence in yak reproductive tissues on the plateau.

The results of immunohistochemistry (Figure 7, Figure 8 and Figure 9) showed that RXRα, RXRβ and RXRγ were positively expressed in the ovaries, fallopian tubes, and uterus of yak at different reproductive stages. In the ovary, RXRα, RXRβ, and RXRγ proteins are mainly expressed in the follicular granular layer (SG), theca follicule (TF), germinal epithelium (GE), and luteal cells (LC). In the fallopian tube, RXRα, RXRβ, and RXRγ proteins are mainly expressed in the mucosal epithelium (EP) and lamina propria (LP); in the uterus, RXRα, RXRβ, and RXRγ proteins are mainly expressed in the endometrium (EM) and uterine glands (UG).

#### IF Detection of RXRα, RXRβ, and RXRγ Distribution in Yak Ovaries, Oviducts, and Uteri Across Reproductive Phases

**Figure 10:**
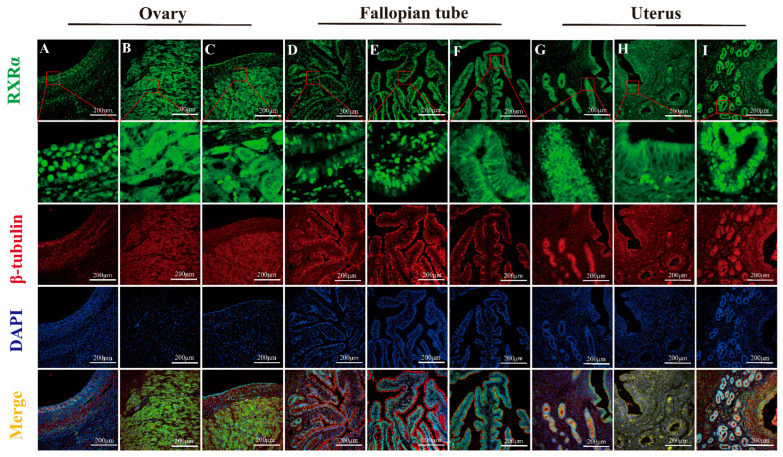
Immunofluorescence staining of RXRα protein in yak ovaries, oviducts, and uteri across different reproductive phases. (**A**–**C**) ovaries; (**D**–**F**) oviducts; (**G**–**I**) uteri. Follicular phase: groups (**A**,**D**,**G**); luteal phase: groups (**B**,**E**,**H**); pregnant phase: groups (**C**,**F**,**I**).

**Figure 11:**
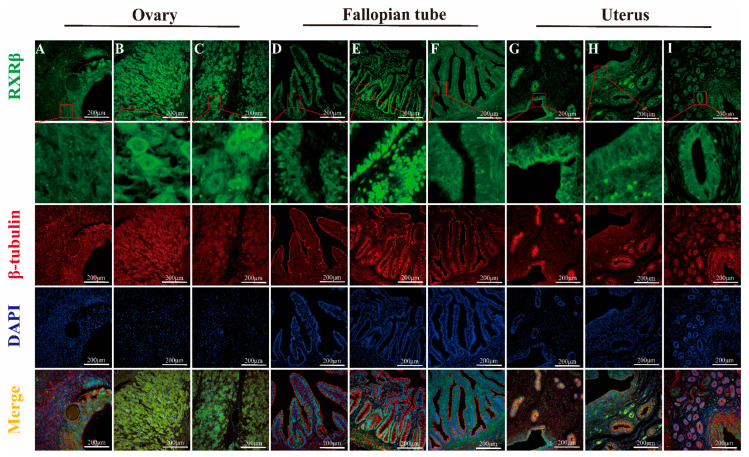
Immunofluorescence staining of RXRβ protein in yak ovaries, oviducts, and uteri across different reproductive phases. (**A**–**C**) ovaries; (**D**–**F**) oviducts; (**G**–**I**) uteri. Follicular phase: groups (**A**,**D**,**G**); luteal phase: groups (**B**,**E**,**H**); pregnant phase: groups (**C**,**F**,**I**).

**Figure 12:**
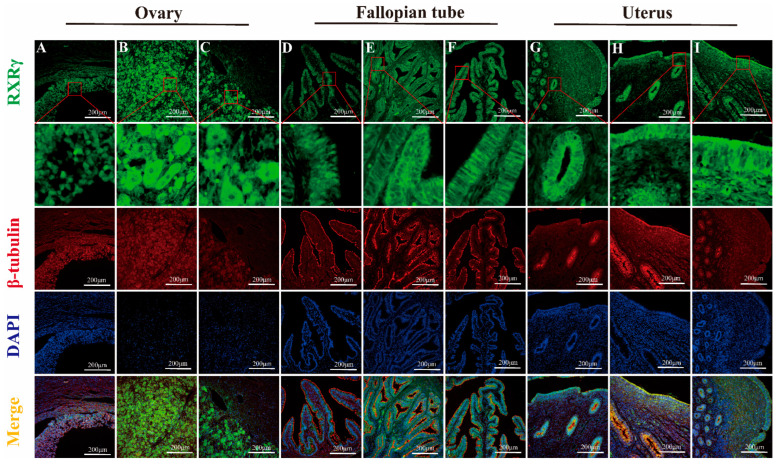
Immunofluorescence staining of RXRγ protein in yak ovaries, oviducts, and uteri across different reproductive phases. (**A**–**C**) ovaries; (**D**–**F**) oviducts; (**G**–**I**) uteri. Follicular phase: groups (**A**,**D**,**G**); luteal phase: groups (**B**,**E**,**H**); pregnant phase: groups (**C**,**F**,**I**).

The results of fluorescence detection showed that RXRα protein was expressed in the nucleus of granulosa cells and theca cells in ovary (Figure 10A). RXRβ and RXRγ proteins were expressed in both the nucleus and cytoplasm in granulosa cells and theca cells (Figure 11A and Figure 12A). RXRα, RXRβ, and RXRγ proteins were expressed in both nucleus and cytoplasm in luteal cells (Figure 10B, Figure 11B and Figure 12B). In the oviduct, the RXRα protein was expressed in the nucleus and cytoplasm of mucosal epithelial cells during the follicular phase and pregnancy (Figure 10D,F), and it was expressed in the nucleus during the luteal phase (Figure 10E). RXRβ and RXRγ were both expressed in the nucleus and cytoplasm during the three stages (Figure 11D,E and Figure 12D,E). The RXRβ protein was mainly expressed in the nucleus during luteal phase, and the expression was weak (Figure 11E). In the uterus, the RXRα protein was expressed in the nucleus and cytoplasm of endometrial cells and glandular epithelial cells in the follicular phase and luteal phase (Figure 10G,H), and it was expressed in the nucleus during pregnancy (Figure 10I). RXRβ and RXRγ proteins were expressed in the nucleus and cytoplasm at all three stages (Figure 11G–I and Figure 12G–I).

## 4. Discussion

As an analysis of RXRα, RXRβ, and RXRγ mRNA and protein levels in yak ovaries, oviducts, and uteri across various reproductive phases, this study’s examination demonstrates significant variation and provides detailed insights into the potential roles of these molecules in yak reproduction—particularly under the harsh, high-altitude conditions of the plateau. Expression levels exhibit variation: they are higher in the ovaries during the follicular phase, then the oviducts seem more active during the luteal phase, and the uteri ramp up when pregnancy hits; indicates that each part is set up in its own way, even though exactly why these shifts occur still remains a bit murky. Some researchers even suspect that things like specific post-translational modifications such as methylation, phosphorylation, or ubiquitination of RXR mRNA during translation—or potentially due to spatial and temporal factors—are behind these patterns, while the focus here stays on protein expression, providing a partial yet informative understanding of RXR’s biological roles. When considering the ovary’s dual role—as both the nurturing ground for egg development and a key endocrine organ—we found that RXRα, RXRβ, RXRγ proteins are ever present in yak ovaries across all reproductive stages. Their expression reaches an evident peak during the follicular phase, significantly overshadowing levels during the luteal and pregnancy phases, and provides compelling insights into their pivotal role in yak reproductive physiology on the plateau. We employed IHC to detect RXRα, RXRβ, and RXRγ proteins in yak ovaries, locating them in follicular granulosa layers, theca folliculi, reproductive epithelia, and luteal cells—findings that echo observations in sheep hypothalami, pituitary glands, ovaries, uteri, and oviducts [18]. It is evident that most ovarian follicles never reach maturity, with over 99% undergoing atresia during estrus [19]. Research findings suggest that RXRs, possibly through PPAR pathways, regulate follicular glucose–lipid metabolism and homeostasis, favoring dominant follicle growth while weaker ones disappear. Notably, RXRα/PPARγ heterodimer levels, detected in primary, secondary, and mature follicles, increase as development progresses [19]. During gonadal differentiation, research findings have shown that the downregulation of RXRγ expression will lead to an increase in the quantity of Sertoli cells and significantly impact the expression of key genes such as Dmrt1 and Sox9 [20]. Dmrt1 and Sox9 are recognized as regulatory genes closely related to follicular development and gonadal differentiation. Such a mechanism provides direct evidence of RXRγ’s participation in the regulation of follicular activation and development. In this study, we found that RXRγ exhibited high expression in the ovaries of yaks during the follicular phase, which provided further evidence that it may play a key role in follicular development and gonadal differentiation through the above pathways. In addition, the function of RXRβ may be related to the synthesis of steroid hormones. Research findings have confirmed that the expression levels of StA directly determine the synthesis efficiency of steroid hormones, and that it serves as a core regulatory molecule in ovarian steroid hormone synthesis [21]. It is speculated that RXRβ may indirectly impact steroid hormone synthesis by regulating the expression of StAR and subsequently participate in reproductive-related endocrine regulation.

The corpus luteum, an endocrine-like gland formed from ruptured follicles post-ovulation, produces progesterone, estrogen, and other hormones to regulate estrus and sustain pregnancy [22]. The results of earlier studies on pseudopregnant canine corpora lutea, using transcriptomics, indicate that LXR-RXR pathways modulate luteal cell lifespan [23]; in comparison, RXRα forms heterodimers with PPARγ, inducing 3β-HSDI transcription to enhance progesterone synthesis [24]. During pregnancy, sustained luteal progesterone levels support gestation, and our findings of positive RXR expression in yak luteal cells, with elevated levels in the ovaries of pregnant yaks, suggest their involvement in maintaining pregnancy, a hypothesis that merits further exploration.

Oviducts serve as the critical site for egg collection, transport, fertilization, and early embryonic cleavage. We found significant RXRα, RXRβ, and RXRγ protein differences in yak oviducts across the different phases, peaking during the luteal phase, with IHC showing their primary locations in the mucosal epithelium and stroma. Ciliated cells in the mucosa drive material transport, whereas secretory cells release substances that create a stable environment for fertilization and early embryogenesis [25]. Post-ovulation, yak oviductal ciliated cells lengthen and multiply, and secretory activity intensifies, facilitating egg movement [26]. Drawing on these findings, RXRs likely support egg and sperm transport and early embryonic development, a notion reinforced by immunofluorescence analysis demonstrating RXRα’s shift from nuclear to cytoplasmic expression in pregnancy, possibly linked to oviductal epithelial apoptosis via Nur77-RXR pathways, which trigger mitochondrial changes and cell death [27,28,29,30]. The results of this study demonstrate that RXRβ exhibited the highest expression during the luteal phase in the oviduct, which differed from the highest expression noted during the estrus period in pigs [31], which may be related to the unique physiological characteristics of yaks. The expression of RXRβ is closely related to estrogen levels, suggesting that its function in the oviduct may be finely regulated by hormone signals. The role of RXRβ in the oviduct mainly includes the migration of oocytes and the maintenance of the fertilization environment. We found that RXRγ was expressed in the mucosal epithelium and lamina propria of the yak oviduct, and the expression levels during follicular phase, luteal phase, and pregnancy were significantly different. At present, there are no reports on its role in the oviduct, combined with distribution, expression characteristics, and RXR family function, it is speculated that RXRγ may play an important role in the secretory function of oviduct epithelial cells by regulating gene expression, metabolism, signaling pathways, and cell differentiation. The authors of future studies can explore in further depth the specific mechanism of RXRγ in oviduct epithelial cells and its potential application value in reproductive health.

In yaks, the uterine endometrium begins as a single layer of flat epithelium during the follicular phase, with ovarian estrogen driving proliferation and glandular development; luteal-phase progesterone then halts this growth, altering its phenotype and function to prepare for embryonic implantation [32]. We found RXRα, RXRβ, and RXRγ proteins abundant in yak uteri across phases, peaking sharply during pregnancy, far exceeding follicular and luteal levels, with IHC revealing their presence in the endometrial epithelium, glands, and vessels, intensifying during the luteal and pregnancy phases. Researchers surmise that these receptors play key roles in early embryonic development and pregnancy maintenance, though whether other mechanisms regulate RXRs in yak organs across phases—and their precise pathways—remains an open question, necessitating further in-depth analysis. Uterine epithelial cells secrete growth factors and cytokines, expressing various receptors to drive proliferation, differentiation, and environmental balance, supporting implantation and gestation [33]. During pregnancy, uterine vessel dilation and increased blood flow enhance maternal–fetal gas exchange and nutrient delivery for fetal growth [34]. With strong RXR expression around the uterine epithelium, glands, and vessels, RXRs likely regulate uterine development and vessel expansion across yak reproductive phases, consistent with findings in cattle [35] and pigs, wherein RXR expression declines with gestational age [36]. The results of studies involving sheep and mice suggest that RXRs, particularly RXRα, are vital for implantation and decidualization, potentially via PPARδ-RXRα heterodimers [18,37], and RXRα’s nuclear presence in pregnant yak endometrial and glandular epithelia indicates its role in sustaining pregnancy, possibly through antioxidant pathways such as protein kinase C, mitigating oxidative stress in vascular endothelial cells [38]. The RXRβ subtype is mainly involved in lipid metabolism pathways and signal transduction [39] and is involved in regulating uterine development and function together with RXRα. Researchers have found that FABP7 and the RXRβ pathway promotes cell survival and proliferation in triple-negative breast cancer [40]. Combined with our experimental results, it is speculated that RXRβ may play a role in the proliferation and repair of the endometrium. The role of RXRγ subtypes in uterine tissue and physiological function remains unclear. In our experiment, the changing trend of the RXRγ protein during different reproductive cycles is consistent with that of RXRα and RXRβ. It is speculated that RXRγ may be more closely involved in synergistic regulation and complement the functions of other RXR subtypes and receptors.

The expression characteristics of RXR in the different reproductive organs of yaks during different reproductive stages were systematically revealed in this study. Based on these findings, it is expected that regulatory strategies for the RXR signaling pathway will be developed in the future to improve the reproductive efficiency of yaks and promote the sustainable development of plateau animal husbandry. For example, regulating the activity of RXRs through drug or gene editing could improve follicular development and luteal function, helping to increase pregnancy and embryo survival rates.

The authors of future studies should investigate the specific molecular mechanisms of RXRs and their heterodimer partners (such as PPARs) and explore their signal networks in yak ovaries, oviducts, and uteri in combination with functional verification experiments. In addition, considering the special plateau environmental adaptability of yaks, the expression regulation and function of RXRs under hypoxia and alpine conditions may reveal their new role in reproductive adaptation. The integration of omics technology, multi-angle experimental design, and in vivo functional intervention will provide a more comprehensive perspective for elucidating the regulation of RXRs in yak reproductive physiology. Larger cohort studies (n = 6–10 per phase) are required to validate these findings and explore environmental influences, such as hypoxia, on RXR expression. In addition, comparative studies with other bovids could aid in elucidating species-specific RXR roles, enhancing reproductive management across high-altitude species.

## 5. Conclusions

RXRα, RXRβ, and RXRγ genes and their proteins are ever present in yak ovaries, oviducts, and uteri across all reproductive phases. They are predominantly located in ovarian follicular granulosa layers, theca folliculi, reproductive epithelia, luteal cells, oviductal mucosal epithelia, and uterine endometrial glands, each displaying distinct patterns depending on the stage, revealing intriguing shifts over time. These findings suggest that RXRs play a significant role in yak reproduction on the plateau, yet their mechanistic pathways, target genes, and interactions with nuclear receptors, such as PPAR and LXR, require further investigation. The results of this study require further validation in studies involving larger populations to support the development of sustainable animal husbandry on the Qinghai–Tibet Plateau.

## Figures and Tables

**Table 1:** Primer information for *RXRα*, *RXRβ*, *RXRγ* and *β-Actin.*

Primer	GenBank Accession Number	Primer Sequence	PCR Product Lengths (bp)	Melting Temperature
*RXRα*	NM_001304343.1	F: GGTCATCCTGCTGCGAG R: GTTTGAGAGCCCCTTGG	265	60
*RXRβ*	NM_001083640.1	F: CGGTGGGAAAGACAAAG R: GGCAAGGAGGAAAAGTG	252	60
*RXRγ*	XM_005890189	F: CCTTTTCCCATCGCTC R: TGTTCTGGATACTTTTGCTT	298	58
*β-actin*	NM_173979.3	F: CCGTGACATCAAGGAGAA R: AGGAAGGAAGGCTGGAAG	172	60

## Data Availability

The data that support the study findings are available upon request.
